# The Anti-Melanogenesis Effect of 3,4-Dihydroxybenzalacetone through Downregulation of Melanosome Maturation and Transportation in B16F10 and Human Epidermal Melanocytes

**DOI:** 10.3390/ijms22062823

**Published:** 2021-03-10

**Authors:** Yi-Jung Liu, Jia-Ling Lyu, Yueh-Hsiung Kuo, Chen-Yuan Chiu, Kuo-Chiang Wen, Hsiu-Mei Chiang

**Affiliations:** 1 Ph.D. Program for Biotechnology Industry, China Medical University, Taichung 40402, Taiwan; u105301602@cmu.edu.tw (Y.-J.L.); u105306601@cmu.edu.tw (J.-L.L.); 2 Department of Cosmeceutics, China Medical University, Taichung 40402, Taiwan; kcwen0520@mail.cmu.edu.tw; 3 Department of Biological Science and Technology, China Medical University, Taichung 40402, Taiwan; 4 Institute of Translational Medicine and New Drug Development, China Medical University, Taichung 40402, Taiwan; 5 Department of Chinese Pharmaceutical Sciences and Chinese Medicine Resources, China Medical University, Taichung 40402, Taiwan; kuoyh@mail.cmu.edu.tw; 6 Department of Biotechnology, Asia University, Taichung 41354, Taiwan; 7 Center of Consultation, Center for Drug Evaluation (CDE), 3F, No.465, Sec.6, Zhongxiao E. Rd., Taipei 11557, Taiwan; kidchiou@gmail.com

**Keywords:** 3,4-dihydroxybenzalacetone, natural antioxidants, melanogenesis, melanosome maturation, melanosome transport, microphthalmia-associated transcription factor, PMEL17

## Abstract

The biosynthesis pathway of melanin is a series of oxidative reactions that are catalyzed by melanin-related proteins, including tyrosinase (TYR), tyrosinase-related protein-1 (TRP-1), and tyrosinase-related protein-2 (TRP-2). Reagents or materials with antioxidative or free radical-scavenging activities may be candidates for anti-melanogenesis. 3,4-Dihydroxybenzalacetone (DBL) is a polyphenol isolated from fungi, such as *Phellinus obliguus* (Persoon) Pilat and *P. linteus.* In this study, we investigated the effects and mechanisms of DBL on antioxidation and melanogenesis in murine melanoma cells (B16F10) and human epidermal melanocytes (HEMs). The results indicated that DBL scavenged 2,2-diphenyl-1-picrylhydrazyl (DPPH) and hydroxyl radicals, and exhibited potent reducing power, indicating that it displays strong antioxidative activity. DBL also inhibited the expression of TYR, TRP-1, TRP-2, and microphthalmia-related transcription factor (MITF) in both the cells. In addition, DBL inhibited hyperpigmentation in B16F10 and HEMs by regulating the cyclic adenosine monophosphate (cAMP)/protein kinase A (PKA), v-akt murine thymoma viral oncogene homolog (AKT)/glycogen synthase kinase 3 beta (GSK3β), and mitogen-activated protein kinase kinase (MEK)/extracellular regulated protein kinase (ERK) signaling pathways. DBL not only shortened dendritic melanocytes but also inhibited premelanosome protein 17 (PMEL17) expression, slowing down the maturation of melanosome transportation. These results indicated that DBL promotes anti-melanogenesis by inhibiting the transportation of melanosomes. Therefore, DBL is a potent antioxidant and depigmenting agent that may be used in whitening cosmetics.

## 1. Introduction

Melanoblasts are derived from neural crest cells and are present in the skin, hair follicles, and eyeballs. Melanoblasts differentiate into melanocytes and initiate melanin production. Differentiated melanocytes transfer mature melanosomes into the surrounding keratinocytes, followed by melanin synthesis [1,2]. The biosynthesis pathway of melanin is a series of oxidative processes that are catalyzed by melanogenesis-related proteins, including tyrosinase (TYR), tyrosinase-related protein-1 (TRP-1), and tyrosinase-related protein-2 (TRP-2). The initial tyrosine is hydroxylated by L-tyrosinase to L-3,4-dihydroxyphenylalanine (L-DOPA), which is oxidized to DOPA quinone. It is then divided into two synthetic pathways: pheomelanin (a red–yellow soluble polymer), mediated via 5,6-dihydroxyindole, and eumelanin, mediated via benzothiazine and benzothiazole [3,4,5,6].

Melanin not only absorbs UV radiation but also has antioxidant and free radical-scavenging capacity. This is the most important light-protective barrier. Although melanin plays an important role in protecting the skin, continuous UV irradiation may lead to the accumulation of abnormal melanocytes, which cause pigmentation disorders, including melasma, freckles, post-inflammatory melanoderma, and solar lentigo [3,4,7,8]. Keratinocytes communicate with melanocytes using cell–cell contacts and secreted factors that bind to the receptors on the melanocyte surface. Keratinocytes influence melanogenesis through paracrine growth factors and cell adhesion molecules [1]. These biofactors include α-melanocyte-stimulating hormone (α-MSH), endothelin-1 (ET-1), interleukin2 (IL-2), basic fibroblast growth factor (b-FGF), stem cell factors (SCF), nitric oxide (NO), adrenocorticotropic hormone (ACTH), prostaglandins, leukotrienes, thymidine dinucleotide, and histamine [1,4,9,10,11]. When exposed to UVB radiation, keratinocytes induce or secrete various biofactors that stimulate melanocyte biosynthesis [4].

Keratinocytes secrete α-MSH, which binds to the melanocortin 1 receptor (MC1R) on melanocytes to trigger melanin synthesis. α-MSH stimulates adenylyl cyclase (AC), thus increasing the level of cyclic adenosine monophosphate (cAMP), to activate the translocation of protein kinase A (PKA) into the nucleus. It activates microphthalmia-related transcription factor (MITF) through phosphorylation of the cAMP response element binding protein (CREB). MITF plays a key role in melanogenesis and controls the expression of melanogenic enzymes, such as TYR, TRP-1, and TRP-2. The MITF protein also regulates melanosome transport and the melanosomal matrix protein premelanosome protein 17 (PMEL17) [1,4,9,12,13]. In addition, MITF is regulated by the phosphoinositide 3-kinase (PI3K)/v-akt murine thymoma viral oncogene homolog (AKT) and mitogen-activated protein kinase (MAPK) pathways. Activation of cAMP downregulates PI3K, AKT, and glycogen synthase kinase 3 beta (GSK3β) expression, which then activates the phosphorylation of MITF at serine 298 and its binding to the M-box of the TYR promoter. This leads to the expression of TYR, which influences melanogenesis. In contrast, cAMP inhibits AKT phosphorylation at threonine 308 and serine 473, and GSK3β phosphorylation at serine 9 to downregulate melanin synthesis [14,15,16,17]. The activation of cAMP regulates RAS and mitogen-activated protein kinase kinase (MEK), which leads to phosphorylation of extracellular regulated protein kinase (ERK), followed by phosphorylation of MITF at serine 73, subsequently leading to the downregulation of TYR and inhibition of melanin synthesis [18,19,20].

3,4-Dihydroxybenzalacetone (DBL) is a polyphenol compound with a dihydroxybenzene structure that is isolated from fungi, such as *Phellinus obliguus* (Persoon) Pilat and *P. linteus* [21]. DBL has been reported to exhibit antioxidative activities such as 2,2-diphenyl-1-picrylhydrazyl (DPPH)-scavenging activity and ferric reducing power [22]. DBL protects against reactive oxygen species (ROS) and apoptosis in H_2_O_2_-induced PC12 cells [23]. DBL has been reported to attenuate inflammation in acute lung injury in mice through the MAPK and PI3K/AKT signaling pathways [21]. DBL not only prevents Parkinson’s disease through AKT/nuclear factor erythroid 2-related factor 2 (Nrf2)/glutathione pathway [24], but also induces autophagy in human neuroblastoma SH-SY5Y cells [25]. DBL exhibits anti-tumor activities, such as against cancer cell proliferation and apoptosis in HCT116 and PA-1 cells [26,27]. To date, no study has discussed the effects of DBL on hypopigmentation. Therefore, in the present study, we investigated the effect and mechanisms of DBL on antioxidation and melanogenesis. Cultured α-MSH-induced murine melanoma cells (B16F10) and human epidermal melanocytes (HEMs) were used in this study to investigate the effect of DBL on melanogenesis and the related mechanisms, including the expression of CREB, PI3K, AKT, GSK3β, MEK, TYR, PMEL17, and MITF in both the cells.

## 2. Results

### 2.1. DBL Scavenged Free Radicals and Reactive Oxygen Species

**Figure 1:**
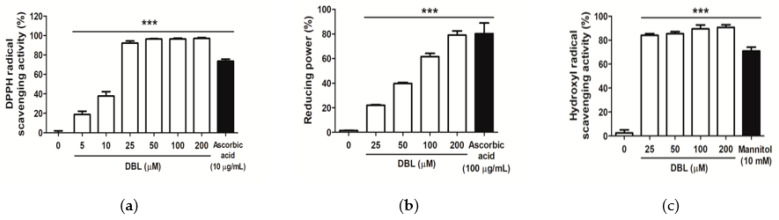
Antioxidative activity (%) of 3,4-dihydroxybenzalacetone (DBL). (**a**) 2,2-Diphenyl-1-picrylhydrazyl (DPPH) radical-scavenging activities, (**b**) reducing capacity, and (**c**) hydroxyl radical-scavenging activities. Each value has been presented as mean ± standard deviation (SD). Significant difference with control group: *** *p* < 0.001.

To investigate the antioxidative effect of DBL, we examined its DPPH radical-scavenging activity, reducing capacity, and hydroxyl radical-scavenging activity. The DPPH radical-scavenging activity of DBL doses over 25 μM was higher than 90%. The DPPH radical-scavenging activity of 50 μM DBL was 92.3%, while that of the positive control, 10 μg/mL (equal to 56.8 μM) ascorbic acid, was 73.7% (Figure 1a). The DPPH radical-scavenging activity of DBL had an IC_50_ value of 11.28 μM.

The reducing capacity of various concentrations of DBL (25 to 200 μM) ranged from 22.0% to 79.1%, while that of the control, 100 μg/mL (equal to 568 μM) ascorbic acid, was 80.3% (Figure 1b). The reducing capacity of DBL had an IC_50_ value of 69.1 μM.

The hydroxyl radical-scavenging activity of various concentrations of DBL (25 to 200 μM) ranged from 84.1% to 90.7%, while that of 10 mM mannitol was 71.0%. (Figure 1c). The results suggested that DBL exhibited strong antioxidative activity. The hydroxyl radical-scavenging activity of DBL had an IC_50_ value of 9.3 μM.

### 2.2. DBL Displays No Cytotoxicity but Reduced Dendrites and Melanin Content in Melanocytes

**Figure 2:**
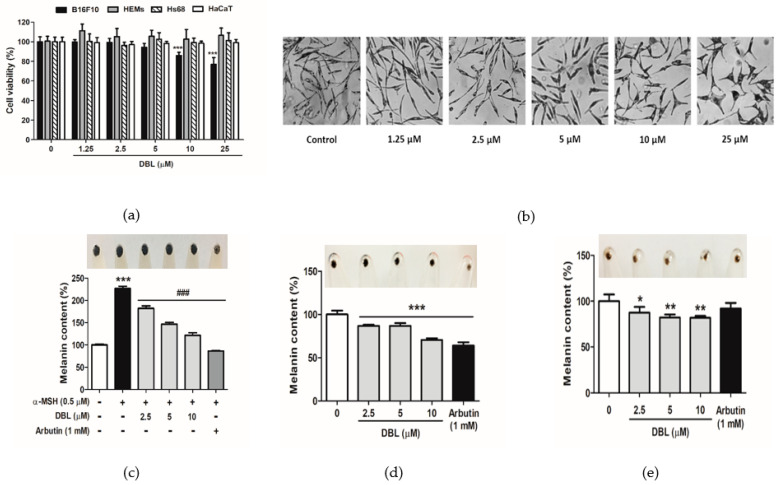
Cell viability, dendritic morphology, and melanin content of DBL. (**a**) The cell viability (%) of B16F10 at 48 h, human epidermal melanocytes (HEMs) at 72 h, and Hs68 and HaCaT cells at 24 h. (**b**) Representative dendritic morphology of DBL in HEMs at 72 h. (**c**) Melanin content (%) of B16F10 and cell pellets stimulated with α-melanocyte-stimulating hormone (α-MSH) and DBL for 48 h. (**d**) Melanin content (%) of B16F10 and cell pellets treated with DBL for 48 h. (**e**) Melanin content (%) of HEM cells and cell pellets treated with DBL for 72 h. All data have been presented as mean ± SD. Significant difference versus control group: * *p* < 0.05, ** *p* < 0.01, *** *p* < 0.001. Significant difference versus α-MSH-treated group: ### *p* < 0.001. Positive control: 1 mM arbutin.

As shown in Figure 2a, upon treatment with 1.25–25 μM DBL, except for 25 μM DBL in B16F10, the cell viability was over 85%; thus, there was no apparent cytotoxic effect of DBL in the B16F10, HEMs, human foreskin fibroblast Hs68 cells, and human keratinocyte HaCaT cells.

The photographs shown in Figure 2b are of HEMs stimulated using various concentrations of DBL (1.25 to 25 μM). HEMs treated with DBL displayed shorter dendritic morphology than the untreated cells. Representative DBL shortened the morphology of dendritic melanocytes so that the melanosomes could neither transfer nor translocate. In this manner, DBL inhibited melanogenesis.

As shown in Figure 2c, the melanin content was 226.8% upon treatment with α-MSH. Following this, when the cells were treated with 2.5–10 μM DBL, the melanin content ranged from 182.4% to 121.4% in B16F10. As shown in Figure 2d, DBL significantly reduced the melanin content in B16F10 without α-MSH. As shown in Figure 2e, the melanin content decreased from 100% to 70.5% after DBL treatment in HEMs.

### 2.3. DBL Reduced Tyrosinase Activity in Melanocytes

**Figure 3:**
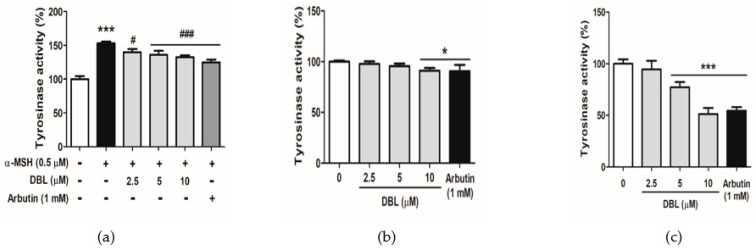
Effect of DBL on tyrosinase activity. (**a**) Tyrosinase activity (%) of B16F10 stimulated with α-MSH and DBL for 48 h. (**b**) Tyrosinase activity (%) of B16F10 treated with DBL for 48 h. (**c**) Tyrosinase activity (%) of HEM cells after 72 h of treatment with DBL. All data have been presented as mean ± SD. Significant difference versus control group: * *p* < 0.05, *** *p* < 0.001. Significant difference versus α-MSH-treated group: # *p* < 0.05, ### *p* < 0.001. Positive control: 1 mM arbutin.

TYR is a key enzyme involved in melanogenesis. Thus, the effect of DBL on TYR activity was evaluated. As shown in Figure 3a, TYR activity was 153.1% after treatment with α-MSH. Following that, when the cells treated with 2.5–10 μM DBL, TYR activity ranged from 140.1% to 132.5% in B16F10. As shown in Figure 3b, DBL also reduced TYR activity in B16F10 that were not induced with α-MSH, ranging from 97.8% to 91.2%. As shown in Figure 3c, TYR activity decreased from 100% to 51.3% after DBL treatment in HEMs.

### 2.4. Effect of DBL on the Expression of Melanogenesis-Related Proteins

**Figure 4:**
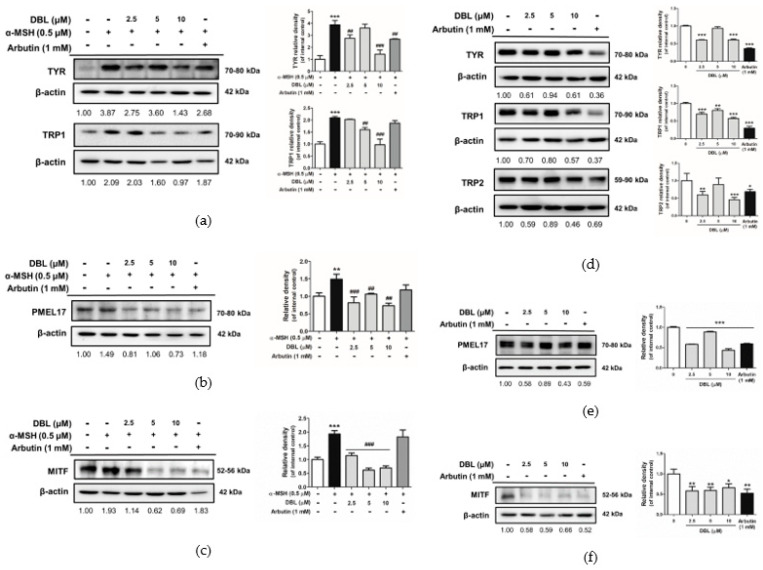
The expression of signaling molecules and melanogenic enzymes, in response to the effect of DBL in α-MSH-induced B16F10 and HEMs. Western blot analysis of the expression of (**a**) tyrosinase (TYR), tyrosinase-related protein-1 (TRP-1), (**b**) premelanosome protein 17 (PMEL17), and (**c**) microphthalmia-related transcription factor (MITF) in DBL-treated and α-MSH-induced B16F10 for 48 h. Western blot analysis of the expression of (**d**) TYR, TRP-1, tyrosinase-related protein-2 (TRP-2), (**e**) PMEL17, and (**f**) MITF in DBL-treated HEMs for 72 h. Inserted values indicated relative protein expression in comparison with β-actin. All data have been presented as mean ± SD. Significant difference versus control group: * *p* < 0.05, ** *p* < 0.01, *** *p* < 0.001. Significant difference versus α-MSH-treated group: ## *p* < 0.01, ### *p* < 0.001. Positive control: 1 mM arbutin.

TYR, TRP-1, TRP-2, and PMEL17 participate in melanin synthesis. As shown in Figure 4a, DBL significantly reduced the levels of melanocytic proteins such as TYR and TRP-1 in B16F10, after stimulation with α-MSH. We investigated whether the effect of DBL on decreasing melanogenesis was associated with melanosome maturation. PMEL17 is an essential protein involved in melanosome maturation. DBL significantly decreased the level of PMEL17 protein in B16F10 stimulated with α-MSH (Figure 4b). All the above melanocytic proteins were involved in melanogenesis and controlled by the MITF transcription factor. Therefore, we evaluated the expression of MITF upon treatment of B16F10 and HEMs with DBL. As shown in Figure 4c, the protein expression of total MITF increased 1.93-fold after treatment with α-MSH. Following that, when the cells were treated with 2.5–10 μM DBL, the expression level decreased from 1.14-fold to 0.69-fold in B16F10. Total MITF protein expression was significantly downregulated by DBL in α-MSH-induced B16F10.

Therefore, we also investigated the expression of TYR, TRP-1, TRP-2, PMEL17, and MITF in DBL-treated HEMs. DBL inhibited the expression of TYR, TRP-1, TRP-2 (Figure 4d), PMEL17 (Figure 4e), and MITF (Figure 4f) in HEMs. The results indicated that the downregulation of melanin synthesis by DBL could be driven by the MITF transcription factor, TYR, and PMEL17.

### 2.5. DBL Inhibited Melanogenesis Through cAMP/PKA, AKT/GSK3β, and MEK/ERK Signaling Pathways

**Figure 5:**
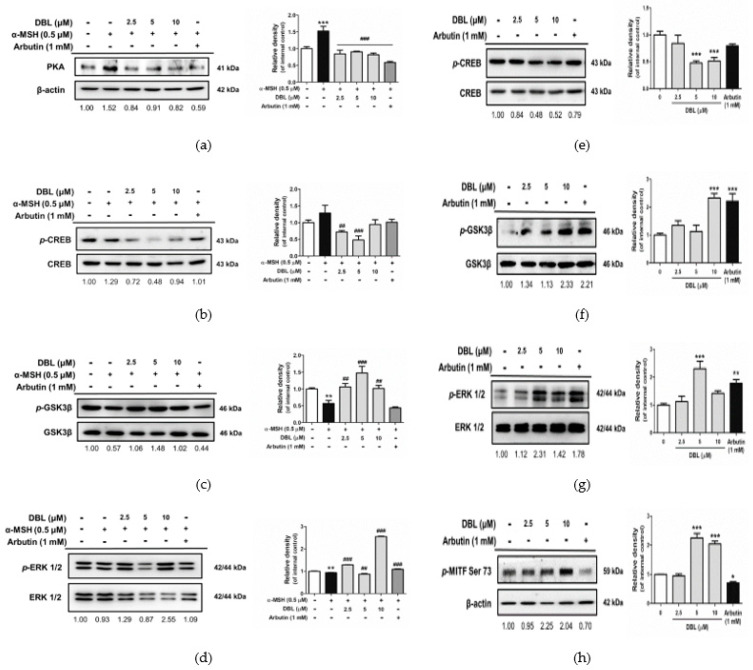
The expression of signaling pathways, in response to the effect of DBL in α-MSH-induced B16F10 and HEMs. Western blot analysis of the expression of (**a**) protein kinase A (PKA), (**b**) *p*-cAMP response element binding protein (CREB), (**c**) *p*-glycogen synthase kinase 3 beta (GSK3β), and (**d**) *p*-extracellular regulated protein kinase (ERK) in B16F10 treated with DBL and induced with α-MSH for 48 h. Western blot analysis of the expression of (**e**) *p*-CREB, (**f**) *p*-GSK3β, (**g**) *p*-ERK, and (**h**) *p*-MITF serine 73 in HEMs treated with DBL for 72 h. Inserted values indicated relative protein expression in comparison with the internal control (β-actin, CREB, GSK3β, and ERK 1/2). All data have been presented as mean ± SD. Significant difference versus control group: * *p* < 0.05, ** *p* < 0.01, *** *p* < 0.001. Significant difference versus α-MSH-treated group: ## *p* < 0.01, ### *p* < 0.001. Positive control: 1 mM arbutin.

Signaling pathways such as protein kinase C (PKC), cAMP, MEK, and wingless-related integration site (WNT) regulate MITF transcription factors, which affect melanin synthesis [12]. To understand how DBL regulates melanin-related proteins, we investigated the effect of DBL on different signaling pathways, including cAMP/PKA, AKT/GSK3β, and MEK/ERK. One of the anti-melanogenic mechanisms is suppression of PKA and *p*-CREB during cAMP signaling. In this study, as shown in Figure 5a, the protein expression of PKA increased 1.52-fold after treatment with α-MSH. Following that, when the cells were treated with 2.5–10 μM DBL, there was a decrease in PKA expression from 0.84-fold to 0.82-fold in B16F10. As shown in Figure 5b, DBL induced *p*-CREB protein expression in B16F10 stimulated with α-MSH. In addition, the AKT/GSK3β signaling pathway is a negative regulator of melanogenesis. Phosphorylation of GSK3β reduces melanogenesis. As shown in Figure 5c, there was a 0.57-fold decrease in the phosphorylation of GSK3β in α-MSH-induced B16F10. Following that, when the cells were treated with 2.5–10 μM DBL, there was an increase in the protein levels from 1.06-fold to 1.02-fold. It is known that activation of *p*-ERK leads to phosphorylation of MITF at serine 73, and subsequent inhibition of melanogenesis [28]. As shown in Figure 5d, the protein expression of *p*-ERK decreased by 0.93-fold in α-MSH-induced B16F10. When these cells were further treated with 2.5–10 μM DBL, there was an increase in protein expression from 1.29-fold to 2.55-fold.

Likewise, as shown in Figure 5e, DBL also inhibited the protein expression of *p*-CREB from 0.84-fold to 0.52-fold in HEMs. In this study, the expression levels of *p*-GSK3β (Figure 5f) and *p*-ERK also increased in DBL-treated HEMs (Figure 5g). As shown in Figure 5h, DBL treatment increased the protein expression of *p*-MITF from 0.95-fold to 2.04-fold in HEMs.

## 3. Discussion

DBL is a polyphenol compound with a dihydroxybenzene structure. It has been reported that plants contain polyphenol compounds that exhibit potent anti-oxidant properties, including tea [29,30], fruits, vegetables, grain products [31,32,33,34], and edible mushroom extracts [35,36]. In addition, the chemical structure of catechols has been reported to have antioxidative and antimelanogenic properties. For example, protocatechuic acid [37], (−)-epigallocatechin-3-gallate [38], ellagic acid [39], and caffeic acid [40,41,42] exhibit excellent antioxidative and antimelanogenic properties. When oxy-tyrosinase brings about oxidation of catechols, the copper atoms of TYR coordinate with the two active site oxygens of catechols [43]. This inactivates TYR, resulting in immature melanosomes and a lack of melanin [44,45]. The present study demonstrated that DBL has potent antioxidative activity, such as DPPH-scavenging activity (Figure 1a), reducing power (Figure 1b), and hydroxyl radical-scavenging activity (Figure 1c). However, a previous study has shown inhibition of DPPH activity and ferric ion reducing antioxidant power upon treatment with DBL [22]. The effect of DBL on DPPH-scavenging activity was stronger in the present study, as compared with that shown in other studies. The IC_50_ of the DPPH-scavenging activity of DBL from *Phellinus linteus* was 11.28 μM (Figure 1a), while a previous study reported that of the DBL isolated from Chaga as 27.75 μM [22]. Several studies have suggested that materials or plant extracts with antioxidative activity may slow down melanogenesis [8,46,47]. In this study, DBL inhibited melanin synthesis in B16F10 and HEMs; this antioxidative activity of DBL may contribute to its anti-melanogenic activity.

Exposure to solar UV directly induces ROS, which may cause oxidative stress, resulting in DNA damage, non-melanoma and melanoma skin cancers, photoaging, and photodermatoses [48,49,50]. Melanin is synthesized by a series of reduction–oxidation reactions that are catalyzed by various melanogenic enzymes such as TYR, TRP-2, and TRP-1 [8,51]. However, another melanogenic protein, PMEL17 (also referred to as gp100, ME20, and a product of the murine silver locus) is a premelanosome protein or pigment transmembrane glycoprotein [52,53]. PMEL induces the maturation of melanosomes from stage I to II through the endoplasmic reticulum [53,54,55] or participates in the biosynthesis of melanin intermediate 5,6-dihydroxyindole-2-carboxylic acid into melanin [56,57]. The melanogenic enzymes TYR, TRP-1, and DOPA chrome tautomerase are enriched in stage III and IV melanosomes [55,58,59]. Watt et al. reported that altering PMEL17 fibrils can result in hypopigmentation [60]. It has been reported that the expression of PMEL17 is dependent on MITF [61,62]. PMEL17 and TRP-2 are unable to express MITF mutations [61]. In our study, DBL downregulated the expression of TYR, TRP-1, and TRP-2, to inhibit melanogenesis. Furthermore, we examined the influence of DBL treatment on the melanosomal matrix protein PMEL17 to further understand the involvement of melanosomes in the pigmentation of DBL. The data showed that DBL inhibited PMEL17, thus inhibiting the maturation and transport of melanosomes, and subsequently melanogenesis (Figure 4b,e). We verified that DBL downregulated the expression of PMEL17 through MITF (Figure 4c,f). MITF has been shown to transcriptionally regulate PMEL17. Our results demonstrated that DBL could shorten the dendritic morphology of HEMs, inhibiting the transfer of melanosomes from melanocytes to the surrounding keratinocytes (Figure 2b). It also reflects the imperfect structure of the dendrites of melanocytes, which hinders the delivery of melanin-synthesizing enzymes, so melanin is not deposited in the melanosomes.

It has been reported that cAMP influences melanin synthesis through the PKA/CREB, MEK/ERK, and PI3K/AKT pathways via α-MSH stimulation [15,63]. Therefore, we investigated the inhibitory effect of DBL on melanogenesis by assessing the regulation of the related signaling pathways. It is known that activation of PKA induces the phosphorylation of the transcription factor CREB and stimulates MITF transcription [64]. Many promoters participate in MITF transcription, including lymphocyte enhancer factor-1 [65,66], CREB [67], Paired Box 3 [68], and SRY-Box Transcription Factor 10 [69]. In particular, binding of phosphorylated CREB to CRE promotes MITF transcription [70]. These data indicate that DBL reduces PKA and *p*-CREB activation in α-MSH-stimulated B16F10 and HEMs. DBL showed stronger effects than arbutin (Figure 5b,e).

The PI3K/AKT pathway plays key roles in melanin synthesis via cAMP-mediated signaling [71]. It has been reported that cAMP promotes the activation of *p*-AKT and *p*-GSK3β through a PI3K-dependent mechanism [15]. It has also been shown that *p*-GSK3β cannot stimulate the action of MITF on the TYR promoter. Furthermore, cAMP has also been shown to induce the activation of RAS and MEK expression, which, in turn, phosphorylate ERK 1/2 (p44/p42 MAPK). Activation of *p*-ERK promotes the phosphorylation of MITF on serine 73, resulting in the suppression of melanin synthesis [15,72,73].

Chao et al. reported that DBL attenuates acute lung injury by suppressing the ROS-mediated MAPK and PI3K/AKT signaling pathways [21]. Gunjima et al. showed that DBL enhances intracellular defense systems against oxidative stress through the PI3K/AKT pathway [24]. Therefore, we hypothesized that DBL could inhibit melanogenesis through the MAPK and PI3K/AKT pathways. We further investigated whether DBL activates or inhibits the MEK/ERK and PI3K/AKT pathways. The results of the present study indicated that not only the downstream protein of *p*-GSK3β but also *p*-ERK is activated by DBL in α-MSH-stimulated B16F10 and HEMs. In addition, activation of *p*-ERK leads to phosphorylation of MITF at serine 73 in DBL-treated HEMs.

However, all of the above pathways suppress or activate MITF expression, resulting in inhibition of melanin synthesis enzymes [74]. MITF is upregulated by CREB and PKA, which are downregulated by the PI3K/AKT and MEK/ERK pathways [12,51,75,76]. In our experiments, DBL significantly reduced *p*-CREB and total MITF expression in HEMs, as well as in B16F10, resulting in decreased TYR, TRP-1, TRP-2, and PMEL17 expression. Additionally, DBL increased the expression of *p*-GSK3β, *p*-ERK1/2, and *p*-MITF (serine 73), inhibiting melanogenesis.

## 4. Materials and Methods

### 4.1. Materials

#### 4.1.1. Chemical Reagents

DBL was synthesized at the laboratory of Professor Yueh-Hsiung Kuo and dissolved in dimethyl sulfoxide (DMSO) for experimentation. The final concentration of DMSO in the medium was below 0.1%. DMSO, DPPH, arbutin, L-DOPA, DL-dithiothreitol, and all other reagents used in this study were purchased from Sigma-Aldrich Chemicals (St. Louis, MO, USA). α-MSH was purchased from Merck (Darmstadt, Germany). All other chemicals used in this study were reagent grade.

#### 4.1.2. Antibodies

Antibodies used in this study, including anti-actin, anti-PKA, anti-GSK3β, anti-*p*-GSK3β, anti-mouse, and anti-rabbit secondary antibody were purchased from GeneTex (Irvine, CA, USA). Antibodies including anti-*p*-ERK and anti-CREB were purchased from Cell Signaling Technology (Beverly, MA, USA). Antibodies including anti-*p*-CREB, anti-MITF, and anti-*p*-MITF were purchased from Abcam (Cambridge, UK). Other primary and secondary antibodies were obtained from Santa Cruz Biotechnology (Santa Cruz, CA, USA).

### 4.2. Antioxidant Assay

#### 4.2.1. DPPH Radical-Scavenging Activity Assay

A previously described method was applied in this study [77]. DPPH was mixed with various concentrations of DBL, added to a 96-well microplate, and incubated at room temperature for 30 min in the dark. Absorbance was measured at 492 nm using a Tecan Sunrise absorbance microplate reader (Sunrise, Tecan, Salzburg, Austria). Ascorbic acid was used as a positive control.

#### 4.2.2. Reducing Power Activity Assay

A previously described method was applied in this study [78] to determine the reducing capacity of DBL. Ferrocyanate and trichloroacetic acid were mixed with various concentrations of DBL. After centrifugation, ferric chloride was mixed with the supernatant and the absorbance was measured at 700 nm. Ascorbic acid were used as positive controls, respectively.

#### 4.2.3. Hydroxyl Radical-Scavenging Activity Assay

Previously used methods were applied to detect the hydroxyl radical-scavenging activity [48]. DBL was mixed with a KH_2_PO_4_–KOH buffer, deoxyribose, FeCl_3_, H_2_O_2_, ascorbic acid, ethylenediaminetetraacetic acid and distilled water. In the next step, 2-thiobrabituric acid and trichloroacetic acid were added to this mixture and incubated at 100 °C for 15 min. After centrifugation, the absorbance of the supernatant was measured at 532 nm using a microplate reader (BioTek, Winooski, VT, USA). Mannitol was used as a positive control.

### 4.3. Cell Cultures and Cell Viability Assay

B16F10 melanoma cells (Bioresource Collection and Research Center, Hsinchu, Taiwan) were cultured in Dulbecco’s Modified Eagle’s Medium supplemented with 10% fetal bovine serum (Gibco, Thermo Fisher Scientific, Waltham, MA, USA). HEMs (ScienCell Research Laboratories, CA, USA) were cultivated in Medium 254 supplemented with 1% human melanocyte growth supplement (HMGS) (Gibco). All cells were maintained at 37 °C in an incubator with 5% CO_2_. The cells were harvested using trypsin. Cell viability was measured using the 3-(4,5-dimethylthiazol-2-yl)-2,5-diphenyltetrazolium bromide (MTT) assay, as previously described [63].

### 4.4. Melanin Content and Tyrosinase Activity Aassay in B16F10 and HEMs

The melanin content and TYR activity of B16F10 were assayed according to a method described in previous studies [79]. Cells, seeded at a density of 2 × 10^4^ cells/well, were cultured in 24-well culture plates and incubated with 2.5–10 μM DBL for 48 h. The absorbance was measured using a microplate reader (Sunrise, Tecan, Salzburg, Austria) at 405 nm for assaying melanin content and TYR activity.

HEMs were cultured at a density of 5 × 10^4^ cells/well in 24-well culture plates. The cells were treated with a medium containing various concentrations of DBL (2.5–10 μM) for 72 h. NaOH (2 N) was added to each well to lyse the cells; following this, the cells were centrifuged. The amount of melanin in the supernatant was spectrophotometrically measured at 405 nm using a microplate reader. For the TYR activity assay, HEMs were seeded at a density of 5 × 10^4^ cells/well in a 24-well plate and treated with a medium containing various concentrations of DBL for 72 h. The medium was removed and replaced with 1% Triton X-100 solution. The mixture was frozen at −80 °C, thawed at room temperature, and then centrifuged. The supernatant was mixed with freshly prepared 15 mM L-DOPA and incubated. The absorbance of each well was measured at 405 nm using a microplate reader.

### 4.5. Western Blot

As previously described [8,13], the expression of melanogenesis-related proteins in B16F10 was detected using Western blot analysis. HEMs (at a density of 1 × 10^5^) were cultured in a 10 cm dish for 48 h. Subsequently, various concentrations of DBL were added to medium 254 + HMGS and incubated for 72 h. Cells were lysed in radioimmunoprecipitation (RIPA) lysis buffer and assessed using the Bradford method (Bio-Rad Laboratories, Hercules, CA, USA). Equal amounts of proteins (30 μg per lane) were subjected to SDS-PAGE, followed by electrotransfer to polyvinylidene difluoride (PVDF) membranes. The blots were blocked with 5% non-fat powdered milk for 30 min at room temperature and then probed with primary antibodies at 4 °C overnight. The membranes were then incubated with secondary antibodies at room temperature for 2 h. Subsequently, the membranes were washed in a 0.1% Tris buffer, and the bands were visualized using an enhanced chemiluminescent reagent and autoradiography.

### 4.6. Statistical Analyses

All results have been expressed as the mean ± standard deviation (SD) of at least 3 independent experiments. For each experimental test condition, significant differences of the samples from their respective controls were assessed using one-way analysis of variance and Tukey’s multiple comparison test. A *p*-value of <0.05 indicated a statistically significant difference.

## 5. Conclusions

**Figure 6:**
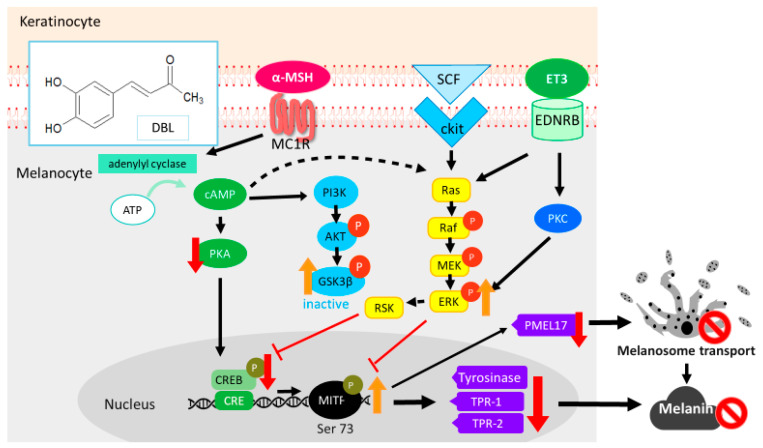
Schematic diagram showing the mechanism of DBL-mediated inhibition of melanogenesis in melanocytes.

This study indicates that DBL modulates melanogenesis through the CREB, MEK/ERK, and PI3K/AKT pathways in B16F10 and HEMs (Figure 6). In addition, it inhibits the maturation and transport of melanosomes by downregulating MITF, TRP-1, and PMEL17 expression. Thus, DBL may be used in lighten hyperpigmentation and developed as a skin-whitening agent and antioxidant.

## Data Availability

Not applicable.

## Abbreviations

α-MSH	α-melanocyte-stimulating hormone
AKT	v-akt murine thymoma viral oncogene homolog
cAMP	cyclic adenosine monophosphate
CREB	cAMP response element binding protein
DBL	3,4-dihydroxybenzalacetone
L-DOPA	L-3,4-dihydroxyphenylalanine
DMSO	dimethyl sulfoxide
DPPH	2,2-diphenyl-1-picrylhydrazyl
ERK	extracellular regulated protein kinase
GSK3β	glycogen synthase kinase 3 beta
HEMs	human epidermal melanocytes
HMGS	human melanocyte growth supplement
MAPK	mitogen-activated protein kinase
MEK	mitogen-activated protein kinase kinase
MITF	microphthalmia-associated transcription factor
PI3K	phosphoinositide 3-kinase
PKA	protein kinase A
PMEL17	premelanosome protein 17
ROS	reactive oxygen species
TYR	tyrosinase
TRP-1	tyrosinase-related protein-1
TRP-2	tyrosinase-related protein-2
